# Heart failure and healthcare informatics

**DOI:** 10.1371/journal.pmed.1002806

**Published:** 2019-05-21

**Authors:** Mohamed S. Anwar, Alan G. Japp, Nicholas L. Mills

**Affiliations:** 1 BHF Centre for Cardiovascular Science, University of Edinburgh, Edinburgh, United Kingdom; 2 Usher Institute of Population Health Sciences and Informatics, University of Edinburgh, Edinburgh, United Kingdom

As biomedical research expands our armoury of effective, evidence-based therapies, there is a corresponding need for high-quality implementation science—the study of strategies to integrate and embed research advances into clinical practice [1]. Large-scale collection and analysis of routinely collected healthcare data may facilitate this in three main ways. Firstly, evaluation of key healthcare metrics can help to identify the areas of practice that differ most from guideline recommendations. Secondly, with sufficiently granular data, it may be possible to detect the underlying drivers of deficiencies in practice. Thirdly, longitudinal data collection should enable us to evaluate large-scale policy initiatives and compare the effectiveness of differing strategies on process and patient outcomes.

## Clinical practice and evidence-based management of heart failure

Heart failure, perhaps more than any other condition, exemplifies the potential for healthcare informatics to bridge the gap between practice and evidence-based care. The prevalence of heart failure is already estimated at 1%–2% and is increasing with our ageing population [2]. Indeed, recent work has demonstrated that incident heart failure cases exceed the four most common causes of cancer combined in the United Kingdom [3]. Moreover, decompensated heart failure accounts for up to 5% of all acute unscheduled hospital admissions and has the longest length of stay of any cardiac condition [4–5]. Given the high rates of debilitating symptoms and death associated with heart failure, this burden to both patients and healthcare systems provides a moral and financial imperative to ensure optimal delivery of proven therapies. The treatment of chronic heart failure has one of the most robust evidence bases in clinical medicine, with multiple landmark trials leading to comprehensive guidelines [6–7]. Yet, despite effective therapies for heart failure being widely available, there is ample evidence to suggest a significant gap exists between guideline-directed practice and clinical practice [8–10].

## Utilising routinely collected healthcare data in heart failure

In *PLOS Medicine*, Rahimi and colleagues report the results of a longitudinal analysis of diagnostic tests, drug prescriptions, and follow-up patterns in 93,000 individuals with heart failure in the UK [11]. The authors show convincingly that some aspects of care, such as the use of diagnostic testing and initiation of evidence-based therapies, have improved over time. Between 2002 and 2014, natriuretic peptide testing was introduced, and the use of echocardiography increased from 17% to 62%. Similarly, the initiation of combination therapy with a beta blocker and angiotensin-converting enzyme (ACE) inhibitor or angiotensin receptor antagonist increased 3-fold. However, other key elements of care, such as dose titration of heart failure therapy, remained poor, and only one in five patients were followed up in primary care, with the rates declining over the study period. A notable strength of the work is the use of both primary and secondary care data within a large and relatively undifferentiated population. This approach ensures that insights from the study are applicable to a general heart failure population and reflect overall care provision. By comparing data across a range of demographic subgroups, the authors hint at factors that may contribute to suboptimal practice. They note deficiencies in a range of quality indicators for women, older patients, and those of low socioeconomic status. It is clear how such findings, if sufficiently robust, could be used to inform future targeted interventions. The study certainly provides us valuable retrospective insights into two major policy initiatives designed to improve standards in heart failure treatment focused on primary and secondary care [11]. The data show persuasively that both the ‘quality and outcomes framework’, a primary care reporting and incentives scheme, and the ‘national heart failure audit’ initiative, a secondary care reporting programme, changed behaviour, but that the outcomes were tied too tightly to specific measures and may have led to unintended consequences. In many respects, it is not surprising that a system designed to reward initiation of heart failure treatments but not subsequent dose optimisation would generate an increase in the former and a decline in the latter. Furthermore, there was a paradoxical decline in the recording of heart failure diagnosis in primary care from 56% to 36%, whereas the diagnosis in secondary care increased steadily. It is possible that practices did not register those patients who would not achieve all management recommendations, in order to maintain high adherence rates. This is a critical lesson to bear in mind whenever policies are geared to improve surrogate markers of quality rather than actual clinical outcomes.

Notwithstanding these strengths, the study does have several limitations. Although it is apparent which components of heart failure care were suboptimal, the data are insufficient in their granularity to detect the drivers for these deficiencies and to evaluate some of the key findings. Specifically, the absence of measures of symptom status, heart rate, blood pressure, left ventricular ejection fraction, comorbidity, and renal function prevent a complete interpretation of the appropriateness of prescribing. Depending on these parameters, initiation or up-titration of heart failure therapies may not have been indicated or may even have been contraindicated. Therefore, the gap between guideline-directed therapy and practice may, in many cases, reflect optimal individualised therapy and sensible clinical judgement rather than systematic deficiencies in care. This point is particularly pertinent to the disparities in prescribing in the elderly, for whom multimorbidity is common. The low rate of follow-up in both primary and secondary care is difficult to interpret in the absence of information from community heart failure programmes, which comprise an essential component of heart failure care delivery. Finally, the insights into the effectiveness of policy initiatives, though interesting, are frustratingly retrospective and in some ways serve mainly to highlight the missed opportunities over the last decade. Moving forward, it is imperative to build a healthcare data infrastructure that is dynamic and can provide insights into contemporary clinical practice.

## Healthcare informatics for the delivery of optimal patient-centred care

**Fig 1:**
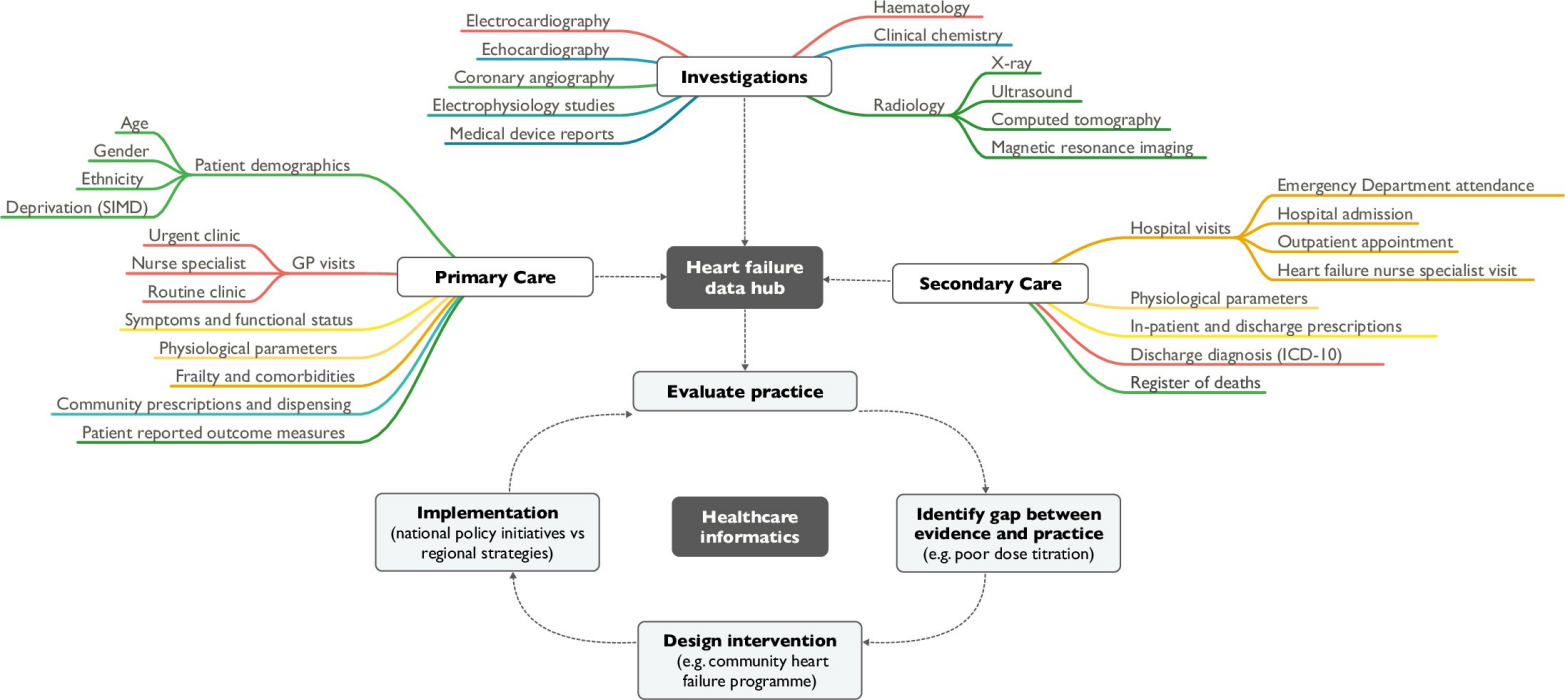
Heart failure data hub and healthcare informatics. GP, general practitioner; ICD-10, International Classification of Diseases-10; SIMD, Scottish Index of Multiple Deprivation.

Our ability to positively impact on current disparities in care are limited because of the absence of comprehensive and contemporary data from across the spectrum of care settings. Insufficiently detailed data impede our ability to identify the causes of disparity in care and, crucially, to determine whether we are providing optimal care on an individualised basis. To successfully overcome these challenges, we need to collate healthcare data from across both primary and secondary care settings in real time and use robust methodology to evaluate major changes in clinical practice or policy decisions [12]. A platform for sharing data between primary and secondary care that is linked, anonymised, and sufficiently granular to facilitate a meaningful evaluation of current practice is required (Fig 1). This approach should be adopted widely in healthcare systems such as the National Health Service to ensure we are providing the highest standards of care for all our patients and using resources most effectively.

## Abbreviations

ACE: angiotensin-converting enzyme
GP: general practitioner
ICD-10: International Classification of Diseases-10
SIMD: Scottish Index of Multiple Deprivation
